# Use of Magnesium Silicate Contaminated with Organic Compounds in Ceramic Materials as a Pore Modifier

**DOI:** 10.3390/ma15248833

**Published:** 2022-12-10

**Authors:** Jolanta Pranckevičienė, Ina Pundienė

**Affiliations:** Laboratory of Concrete Technology, Institute of Building Materials, Vilnius Gediminas Technical University, Linkmenų Str.28, 08217 Vilnius, Lithuania

**Keywords:** magnesium silicate, organic compounds, ceramics, structural parameters, thermal conductivity, reusing

## Abstract

This study investigated the use of organic compound waste (OCW) contaminated magnesium silicate/diatomite in ceramics. Substituting part of the clay (between 5 and 20 wt.%) with OCW modifies a pore structure and enhances the ceramic product’s thermal conductivity, density, and frost resistance. Prepared samples were tested at 1000–1060 °C temperatures and their structural parameters and Maage factor, useful for frost resistance prediction, were evaluated. Results show that OCW modifies the porous structure and improves the insulating properties of the ceramic body. Increasing OCW content up to 15% in the ceramic body decreases density by up to 15.0%, and thermal conductivity by up to 42.5%, because of the modified pore structure. According to structural parameters calculation, the higher frost resistance can be predicted for ceramic bodies containing 5–10% of OCW, according to Maage factor calculation ceramic bodies containing 5–20% of OCW are frost resistant. Designed ceramic products can be attractive for use in construction due to improved energy efficiency and reduced energy consumption in buildings due to their low thermal conductivity, satisfactory mechanical strength, and sustainability based on predicted frost resistance.

## 1. Introduction

Waste management in developed countries is focused on waste reduction through zero-waste or low-waste technologies, improvement of waste quality parameters, and waste recycling. However, industrial development makes the issues of waste generation and management a great concern. Poor waste management has many negative consequences such as atmospheric pollution and numerous challenges faced by businesses, governments, and society [1,2,3,4]. Technogenic waste application in the production of building materials is one way to increase the effectiveness of using non-renewable energy resources, and to mitigate the negative impact of industry on the environment; thus, ensuring the sustainable use of natural resources. The use of technogenic waste in the manufacture of lightweight materials could contribute to solving the challenge of energy efficiency in the construction sector. In the manufacture of ceramic materials, one of the benefits of using technogenic or agricultural waste is the energy costs reduced during the burning process and the possibility of creating porous or microporous structures. Organic waste produces extra energy during combustion. Research has shown that the weight of up to 10 percent of organic waste can ensure an appropriate enhancement in the porosity and thermal conductivity of ceramic lightweight bricks [5].

Technogenic and organic natural waste can be successfully employed in the manufacturing of various construction materials [6,7,8,9,10,11], especially in the ceramic industry, via the production of high-quality ceramic products [12,13,14,15,16,17,18,19,20,21]. The use of pore-forming substances in ceramic materials is an innovative direction for the recovery of waste from agriculture and serving industries, as the formation of pores during the burning process will improve the properties of ceramic products positively [5,22,23]. Previous reports tested the burning of olive mill residues for obtaining a porous structure in a ceramic body [22,24,25]. The porosity of ceramic bodies fired at 950 °C containing 10% olive mill residues increased from 27.8 to 44.9%; however, the compressive strength was reduced by more than three times, from 36.9 to 10.26 MPa. Using higher amounts (up to 23 wt.%) of the same residues drastically decreased the mechanical properties of ceramic bricks [26,27]. Porous structure formation was tested in anorthite ceramics through the combustion of paper-processing residue as an additive [25]. The ceramic body had a porous structure and demonstrated very high compressive strength values (8–43 MPa); however, a high firing temperature from 1200–1400 °C is required to achieve these values. Firing is a highly important process in the manufacturing of ceramic products with low-cost and combustible residue types (organic, mineral, or mineral with organic compounds), due to the potential to reduce the temperature required for the sintering process and lower the energy costs of production [28,29]. Many research papers are describing the positive effect of technogenic waste on the physical and mechanical properties of ceramic products [30,31,32,33,34,35].

Many reports are devoted to investigating the impact of technogenic waste on the increase in the porosity of ceramic products [5,36,37,38,39]. Research has shown that diatomaceous residues from the agro-crop processing plant, or diatomaceous filter waste from breweries, can be successfully integrated into the composition of ceramic products [33,40]. Moreover, widely used as pore-creating additives are alkaline earth carbonates (calcite, magnesite, and dolomite) [41]. Sintered diatomite has been successfully used as a porous ceramic membrane for microfiltration [15,42]. Diatomite is a sedimentary rock resulting from the siliceous fossilized skeletons of diatoms, which are composed of rigid cell walls named frustules [43,44,45]. It was reported that diatomite-based materials allow management over the pore size modifying and mechanical properties improving [43,46]. Such rocks are minerals with a large variety of attractive features. As a consequence, they are extensively used in the industry. The presence of amorphous active silicon dioxide, and also a finely porous structure, lightness, and low thermal conductivity are their essential characteristics. These properties render these materials intensely chemically active and allow using them as sorbents, desiccants, catalysts, materials for filtration and insulation, as well as catalyst and filler carriers. The size of pores and pore walls of diatoms are mostly nano-sized, which confirms their classification as nanomaterials [43].

One of these most promising mineral residues is diatomaceous earth with adsorbed organic compounds, from paraffin wax production. A paraffin wax production line with yearly productivity of 24,000 tons produces about 2300 tons of paraffin wax feedstock, which consists of diatomaceous earth and organic compounds. The annual paraffin wax output in Europe is over 750,000 tons [47]. The most common paraffin wax production technology is the dewaxing of light lubricating oil stocks to the required paraffin wax quality level, and then forming the final product into blocks or granules. Purification is done in reactors by filtering the feedstock through the adsorbent–diatomaceous earth is used as a filter media layer. The paraffin wax manufacturing process generated waste (OCW) is regularly removed from the system and transported to the landfill. OCW is a sort of residue that, because of its composition, the volume produced, and the existing absence of clear solutions for recovery, is complicated to handle and manage, as there are no real purification alternatives because of the expensive and ineffective cleaning and recycling process. OCW is a prospective technogenic additive that can be applied in ceramic product manufacturing to increase porosity and enhance the sustainability and insulating properties of fired ceramics. As indicated in a previous study [33], diatomaceous residues are known to decrease energy expenditure in the production process due to the energy emitted during the burning process, resulting from the burning of the residue’s organic matter. Diatomaceous earth is light and porous has a large specific surface area and has excellent potential for absorption. It is also characterized by low thermal conductivity and a high melting point [15,48,49]. Application of diatomaceous earth in the production of bricks [50] and hollow stones is known, as a raw brick with this additive does not fissure and distort after fast drying; this additive results in small shrinkage during firing, without causing loss of strength. As reported in research [51] scientists fired specimens containing diatomaceous earth and agro-waste ash at temperatures below 960 °C and obtained a ceramic body whose density and porosity do not exceed 1100 kg/m^3^ and 50%, but also with low compressive strength (8.5 MPa).

Diatomaceous earth containing certain clay minerals can be used in the production of low-density products, such as lime and gypsum mix [50]. The density of these products ranges from 730–880 kg/m^3^ and compressive strengths range from 14.5–17.5 MPa. In another report, it was proven that for the production of bricks with satisfactory insulating properties diatomaceous filtration residues from the oil refinery plant can be utilized. The mechanical properties of brick did not essentially deteriorate when the content of residues did not exceed 10 percent [40]. Additionally, it was concluded that diatomaceous filtration residues decrease energy consumption during the brick production process, as during the burning process organic compounds, presented in diatomaceous filtration residues additionally produce energy and lower ceramic body sintering temperature [33].

The present research deals with little-explored diatomaceous earth residues from paraffin wax manufacturing (OCW), used as an alternative, economically reasonable as pointed out in [33], value-added technogenic waste material in the development of porous ceramics with enhanced porosity and thermal conductivity. In contrast to the studies conducted by [33] in which diatomaceous residues were limited to only 10 percent, in this study the OCW amount has been increased to 15 percent in the ceramic body. The thermal analysis of OCW alone and the ceramic body with OCW was done in purpose to reveal the effect of OCW addition on the firing process of the ceramic body.

This study revealed the effects of OCW on physical and mechanical properties, open porosity, and pore size distribution, and determines structural parameters and Maage factor, which allows predicting the frost resistance, very relevant to northern climatic conditions of the ceramic body. These calculations are very important because ceramic body sustainability depends most on frost resistance. In the study, established microstructural and thermal properties significantly enrich the application of developed materials in construction ceramic products.

## 2. Materials and Methods

For this investigation, illite clay, sand (fraction 0–1 mm), and diatomaceous earth residues from paraffin wax manufacturing (OCW) were used. OCW was milled in a laboratory mill. OCW is light, with a bulk density of 440 kg/m^3^. As-received clay was dried at 105 ± 5 °C, and additionally ground in a laboratory ball mill and sieved through a 0.63 mm sieve. The reference forming mass composition (CS), consisting of dry clay and sand, and three other compositions (Table 1) containing OCW in amounts of 5, 10, 15, and 20 wt.%, named CD5, CD10, CD15, and CD20 respectively, were prepared in a special planetary mixing device for 20 min to reach a homogenous dry mixture. It is noted that OCW is easily mixed with clay.

After mixing, molding masses were wetted (~20–22%) until a consistency suitable for the molding mixture was obtained. The prepared mass was conditioned for 3 d at 95 ± 5% humidity to reach an even distribution of the humidity in the forming mixture. After 3 d, 70 × 70 × 70 mm samples were prepared. Thirty-two specimens were prepared for each forming mixture. For each firing temperature, 8 specimens were used. The samples were held under common laboratory conditions for 3 weeks and then dried to a constant mass at 105 ± 5 °C, under the following procedure: drying in the oven at 60 ± 5 °C for 1 day and drying in the oven at 105 ± 5 °C for 1 day further. Dried specimens were fired at 1000, 1020, 1040, and 1060 °C. The overall firing duration is 36 h, maintaining the highest firing temperature for 8 h.

Using Oxford Instruments INCA PentaFET3, the chemical composition of clay, sand, and PMV was established. The organic compounds present in OCW were analyzed by gas chromatography (Shimadzu QP2020). In compliance with the appropriate standard test methods, the key features of ceramic samples were tested: EN 772-13 for density testing; EN 771-21 for water absorption (Ws) testing; and EN 772-1 for compressive strength testing. The content of organic impurities in OCW was measured according to EN 13820. A Cilas 1090 L analyzer was employed to examine the particle size distribution in clay, sand, and PMV. Dilatometry tests, adhering to a controlled heating rate of 4 oC/min, were performed using a Linseis L76 unit. Differential thermal analysis was done by thermogravimetric analytical instrument (Linseis STA PT-1600), adhering to a controlled heating rate of 10 ºC/min. X-ray tests were done using an X-ray diffractometer (SmartLab, Rigaku). Ultrasonic tests of ceramic bodies after firing were done using a PUNDIT-7 tester. Thermal conductivity was measured using a LaserComp FOX 304 tester, according to EN 12667:2002. The distribution of pores in the ceramic sample was examined by adapting a Pore Master PM33-12 device, and the sample for testing was taken from the internal layer of the specimen. Applying a scanning electron microscope (SEM, JEOL JSM-7600F) the microstructure of samples was examined. The apparent porosity of ceramic bodies was tested according to EN ISO 10545-3. Two potential ceramic body frost resistance prediction methods were used in this study. The first method is the one developed by Maage [52,53], in which the potential frost resistance is evaluated with a single numerical quantifier, determined according to the following formula:

Fc = 3.2/PV + 2.4 × P3,(1)

where Fc—frost resistance number, PV—pore volume in cm^3^/g, and P3—a percentage of the pores with diameters larger than 3 µm.

Another method is based on the estimation of the parameters of structure–effective porosity (W_e)_, apparent porosity (W_r)_, reserve pore volume (R_p_), and relative wall thickness of pores and capillaries of ceramic bodies (D). These structural parameters have been defined in compliance with the procedure described in [54,55]. The effective porosity of a ceramic body is defined as the number of pores and capillaries that are efficiently working. Apparent porosity is defined as the total open porous volume of the ceramic sample, in both macrostructure and microstructure dimensions. Reserve of the pore volume is defined as the number of reserve pores and capillaries scarcely entered by water or plastic ice. The larger the reserve of pore volume, the higher the exploitation of frost counteraction of ceramics. The relative wall thickness of pores and capillaries is defined as the qualified thickness of the pore and capillary walls. The greater thickness of the pore and capillary walls, the greater the exploitation of frost counteraction of products.

To assess the temperature, when the viscosity of the softened ceramic body, containing SiO_2_, Na_2_O, CaO, MgO, and Al_2_O_3_ reach the 10^2^–10^12^ Pa·s range, the calculation described in the research was employed [56]:

T_ƞ_ = K_1_[Na_2_O] + K_2_[CaO + MgO] + K_3_[Al_2_O_3_] + K, (2)

where:

T_η_: is the temperature corresponding to the target viscosity, Pa·s (lg η = 10^9^),

K_1_, K_2_, K_3_, K: selected constants for calculating temperatures corresponding to the target viscosity.

The modulus of acidity and melting capability modulus were calculated per [57,58,59]. Acidity modulus is the ratio of acidic oxides to the basic oxides as represented in the equation:

M_a_ = mSiO_2_ + mAl_2_O_3_/mCaO + mMgO, (3)

where:

M_a_: modulus of acidity,

m: mass content of oxides (wt.%).

The equation relies on the weight percentage of the included oxides. The melting capability modulus is defined as the ratio of the molar fractions of the acidic oxides to all other oxides.

M_k_ = m(SiO_2_ + Al_2_O_3_ + Fe_2_O_3_ + TiO_2_)/m(CaO + MgO + Na_2_O + K_2_O), (4)

where:

M_k_: the melting capability modulus,

m: mass per cent of oxides (wt.%).

## 3. Results

### 3.1. Characterisation of Used Materials

**Figure 1:**
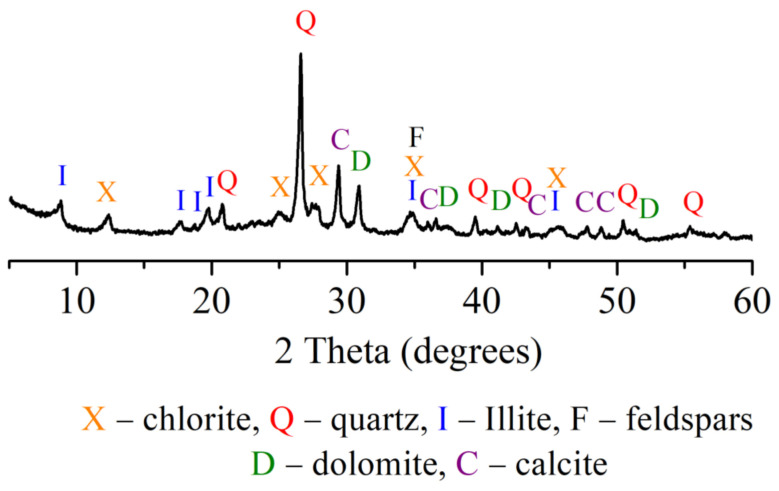
Clay XRD analysis.

**Figure 2:**
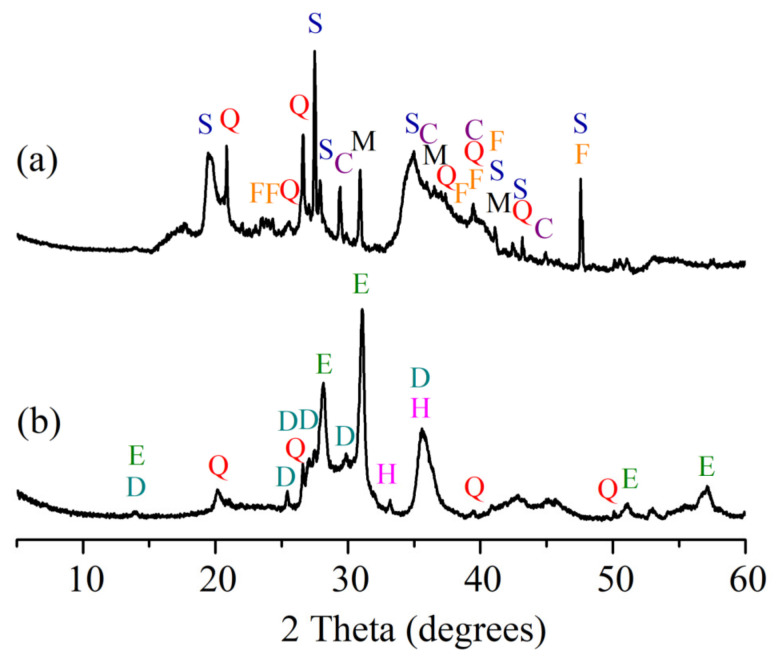
OCW analysis: (**a**) before firing; (**b**) fired at 1000 °C.

**Figure 3:**
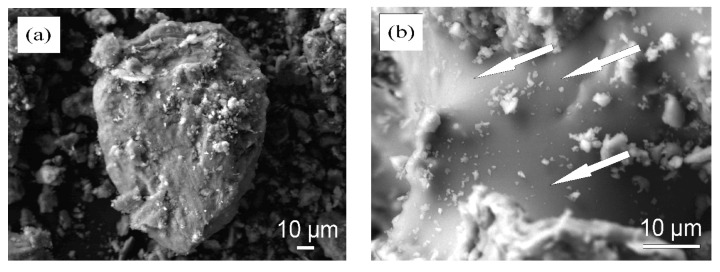
SEM image of (**a**) non-fired OCW and (**b**) OCW after firing at 1060 °C.

**Figure 4:**
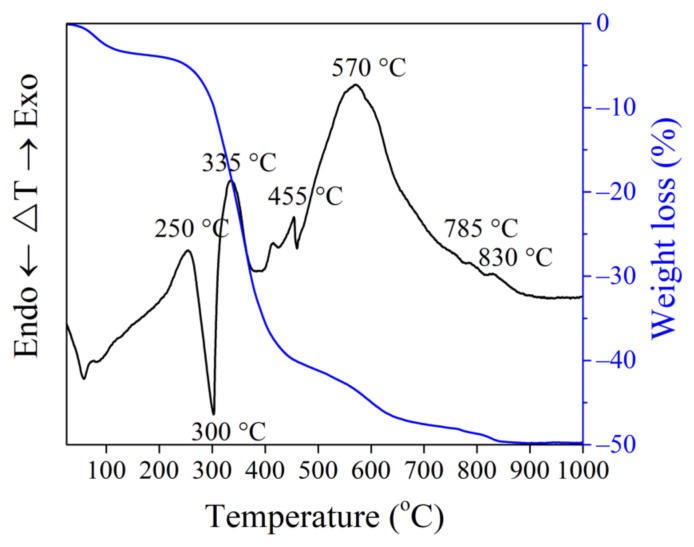
Thermal analysis of OCW up to 1000 °C.

Results presented in Table 2 demonstrate that clay contains coarse carbonaceous impurities, and the Al_2_O_3_ and TiO_2_ content is 16.0%. (Table 2). Chlorite ((Mg,Fe)_4_(Al,Fe)_2_(OH)_8_Al_2_Si_2_O_10_), illite (K,H_3_O)(Al,Mg,Fe)_2_(SiAl)_4_O_10_[(OH)_2_,H_2_O], feldspars (NaAlSi_3_O_8_), quartz (SiO_2_), calcite (CaCO_3_), and dolomite (CaMgCO_3_) (Figure 1) represent the mineral composition of the clay. The tests revealed the prevalence of SiO_2_ (27.4%), MgO (14.0%), CaO (3.5%), alkali oxides (Na_2_O and K_2_O) (4.9%), and traces of Fe_2_O_3_, Al_2_O_3_ and loss on ignition reached 46.8% in the OCW. The mineral composition of non-fired OCW is as follows: quartz (SiO_2_), calcite (CaCO_3_), sepiolite (Mg_4_(OH)_2_[Si_6_O_15_]_6_H_2_O), forsterite (Mg_2_SiO_4_), magnesium silicon oxide hydroxide hydrate (Mg_3_Si_4_O_10_(OH)_2_(H_2_O)) (Figure 2). After firing at 1000 °C, the crystallization of the following minerals is observed: enstatite (MgSiO_3_), with the most intense peaks, followed by diopside (CaMg[Si_2_O_6_]), hematite (Fe_2_O_3_), and quartz (SiO_2_). Some crystals, however, remain in an amorphous phase (Figure 2). The OCW particles’ microstructure is presented in Figure 3a. Microstructure tests revealed that firing at 1060 °C induces the formation of the liquid phase where OCW particles melt (Figure 3b). The differential thermal analysis (DTA by TG) curves for OCW up to 1000 °C (Figure 4) indicate a broad endothermic effect at 60–100 °C, correlated with the removal of unbound water with mass loss of 5.8%. This OCW thermal degradation showed a multistage process with four exothermic peaks at 250, 335, 455, and 570 °C. Mass loss arises from different organic compounds of OCW, with overall mass loss reaching 42%. Volatile components of paraffin decomposed first, followed by heavier compounds [5]. OCW completely combusted at 570–590 °C. Two small endo-effects at 785 °C and 830 °C arise from the decarbonation process of CaCO_3_ and MgCO_3_. The loss of mass during the decarbonation process reached 2.5%.

**Figure 5:**
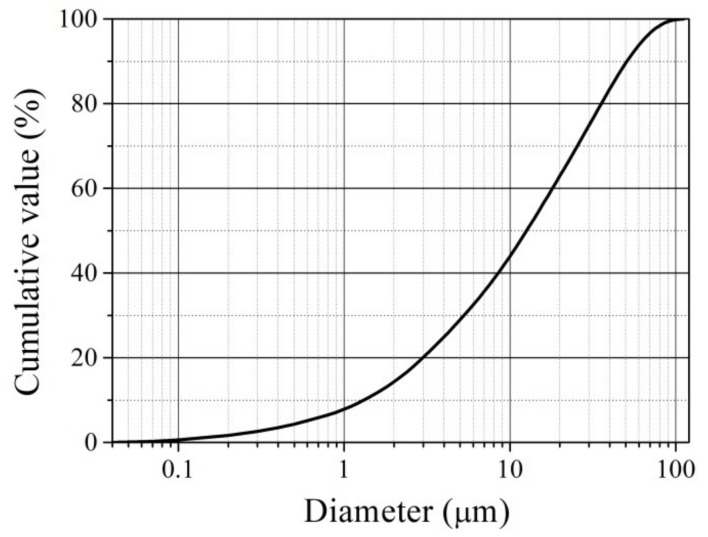
OCW particle size distribution.

OCW particle size ranges from 0.04–112 µm, with an average size of 20.1 µm (Figure 5). From gas chromatography results, the organic compounds in OCW pose no health hazards; therefore, OCW can be safely used in ceramic product manufacturing. Table 2 illustrates the chemical composition of materials. Chemical compositions of the forming masses CS, CD5–CD20 calculated by eliminating losses on ignition are presented in Table 3.

### 3.2. Thermal Analysis

**Figure 6:**
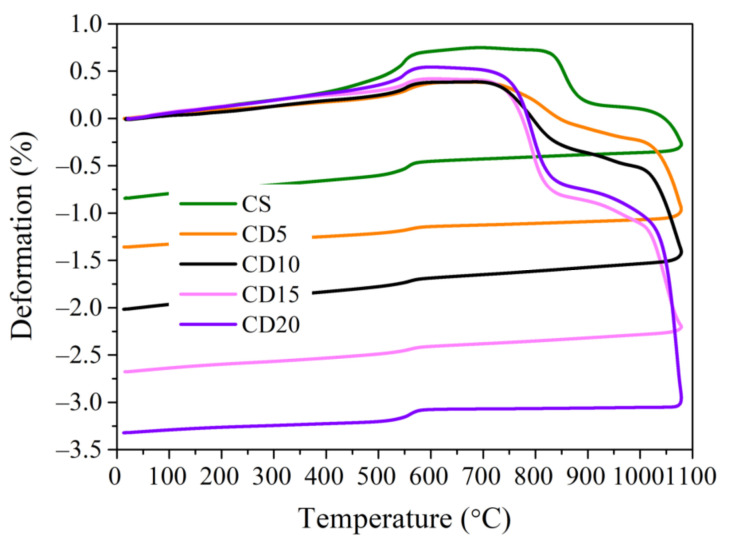
Dilatometric curves of ceramic bodies fired up to 1080 °C.

**Figure 7:**
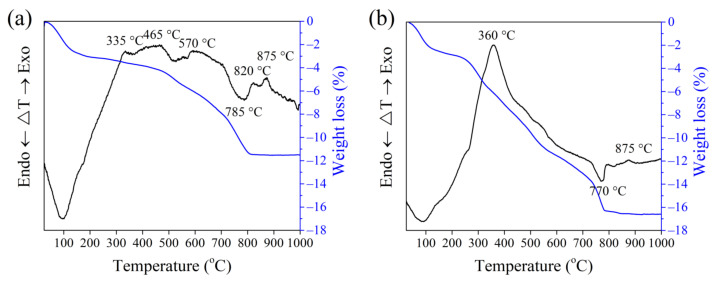
DTA curves of ceramic bodies (**a**) CS and (**b**) CD20, fired up to 1000 °C.

The thermal dilatometric curves (Figure 6) of reference CS and CD5–CD20 compositions, incorporating 5–20 wt.% of OCW, during thermal treatment of up to 1080 °C, and the DTA by TG curves of CS and CD20 recorded up to 1000 °C (Figure 7) show that the first endothermic effect, associated with the release of free water from CS and OCW-containing compositions appears at 90–100 °C. Dilatometric curves of all compositions exhibit a continuous slight expansion during firing until 500–550 °C. However, the expansion of ceramic bodies with increasing OCW amounts gradually decreases, which can be related to the reduction in quartz amount in the compositions with OCW and diatomaceous earth porous structures, which compensate for expansion. This is caused by the fact that the organic compounds present in the diatomaceous waste porous structure are released without breaking the structure. Similar trends using spent diatomite in ceramic bodies were previously observed elsewhere [30,33,34,40,60]. Clear exothermic peaks at 200–450 °C appear in the DTA curves of sample CD20 with the peak value at 345 °C, with the mass loss increasing to 6.5%. The oxidation and/or thermal decomposition of organic compounds existing in OCW causes the occurrence of this exothermic peak. The peak does not appear in the dilatometric curve, possibly due to not having sufficient calorific power [33]. A broad endothermic peak for CS (510–570 °C) and a low-intensity peak for CD20 indicate the division of chemically-connected water at 510 °C [61], and a little endothermic peak at 573 °C indicates the polymorphic modification of quartz [62]. Starting at 530–540 °C, an expansion of ceramic bodies increases up to approximately 600 °C, mostly because of the polymorphic quartz modification, as is seen in the thermal dilatometric curves with a little endothermic peak at 570 °C (Figure 7). Besides quartz polymorphic modification processes, dehydroxylation of the clay mineral illite [63], present in the tested clay, occurs between 500–700 °C, with mass loss of approximately 3.1–3.4% in tested compositions.

At temperatures exceeding 600 °C, expansion gradually loses intensity up to 845 °C in the CS ceramic body, at which point these materials reach their maximum expansion, as shown in Figure 6. For samples with OCW addition, expansion finishes at lower temperatures: for CD5 at 835 °C, for CD10 at 810 °C, for CD15 at 790 °C, and for CD20 at 780 °C. The maximum expansion for composition CS is 0.75%, and that of compositions with OCW is lower. As described previously [33], the maximum expansion is inferior in ceramic bodies with OCW, due to the creation of a more porous structure in this material after the burnt out of its organic compounds. For composition CD20, expansion reaches 0.375%, meaning that OCW addition of 20% reduced expansion deformation by 71%, from 0.75 to 0.215%. Maximum expansion values from 0.75–0.215% are deemed reasonable for ceramic product manufacturing for construction [33]. This is highly important, as during the modification of quartz minerals the ceramic body is not completely developed, and expansion deformations may be given rise to cracking of ceramic structure that can deteriorate the ceramic body’s characteristics. This phenomenon can be justified by the porous structure of diatomaceous earth, and by lower quartz amounts (down to 10%) in ceramic bodies with added OCW [64].

The clearly recognized CaCO_3_ and MgCO_3_ decarbonation endothermic effects coinciding with the onset of the contraction process were observed at 800 °C in the case of CS, and at 770 °C in the case of CD20. Loss of mass during the decarbonation process reaches 2.5–3%. A small exothermic peak appears in the DTA curve with a peak value at 875 °C, due to MgO oxidation. It is also important to highlight that, after thermal treatment up to 1000 °C, mass loss in the CS ceramic body reaches 11.5%, while in the CD20 ceramic body mass loss was higher, reaching 16.5%.

Sharp shrinkage starts at 845 °C and reaches 910 °C for the CS ceramic body. For compositions CD5, CD10, CD15 and CD20 this starts from 790–820 °C until reaching 920–950 °C. The oxides that facilitate an increased viscosity are SiO_2_, Al_2_O_3_, MgO, and Fe_2_O_3_, while alkali metal oxides like K_2_O, Na_2_O, and FeO contribute to decreasing the viscosity at softening temperatures [57]. Due to the higher amount of alkaline compounds (Na_2_O + K_2_O), increasing from 2.57 to 3.37% and alkaline –earth metal compounds such as calcium and magnesium (Table 3) present in this mixture, ceramic bodies CD5, CD10, CD15 and CD20 reach the sintering temperature earlier than CS. These compounds serve as fluxing additives while also causing greater shrinkage. These results are confirmed by the calculations, presented in (Table 4) of acidity modulus (M_a)_, melting capability modulus (M_k_), and temperature (T_η_) at which the ceramic body reaches the same viscosity. The larger the value of (M_a_) of the initial raw material, the higher the viscosity of its melt is. The increase in the alkaline compounds (M_a_) and (M_k_) decreased from 5.66 to 4.89 and from 4.62 to 3.48, and (T_η_) decreased from 843 to 791°C. The calculated temperature corresponds to the trends of dilatometric curves obtained in Figure 6.

When the shrinkage process finishes, a period without material volumetric changes takes place up to 1000–1030 °C for ceramic bodies CD5, CD10, CD15, and CD20 and up to 1050–1060 °C for ceramic body CS.

A shrinkage increase of up to 1080 °C, occurring at a much lower rate, in the CS and CD5 compositions reaches 0.375 and 0.25%, respectively, whereas, in the CD10, CD15, and CD20 compositions, shrinkage at peak temperature reaches 0.75%, 1%, and 1.6%. Shrinkage values up to 2% are common for ceramic compositions used to prepare porous ceramic products. This study shows that OCW is suitable for manufacturing ceramic products, as at temperatures of 800–850 °C the samples are fully expanded, then begin to melt, and these temperatures are typical treatment temperatures in the ceramic industry, where low-melting illite clay is used. An overview of the results of dilatometry and DTA, the temperature range 1000–1060 °C was chosen, as at 1000 °C, and up to 1060 °C, the creation of new phases is observed, because the shrinkage results in dilatometry test confirm softening of ceramic bodies begin.

### 3.3. Microstructure

**Figure 8:**
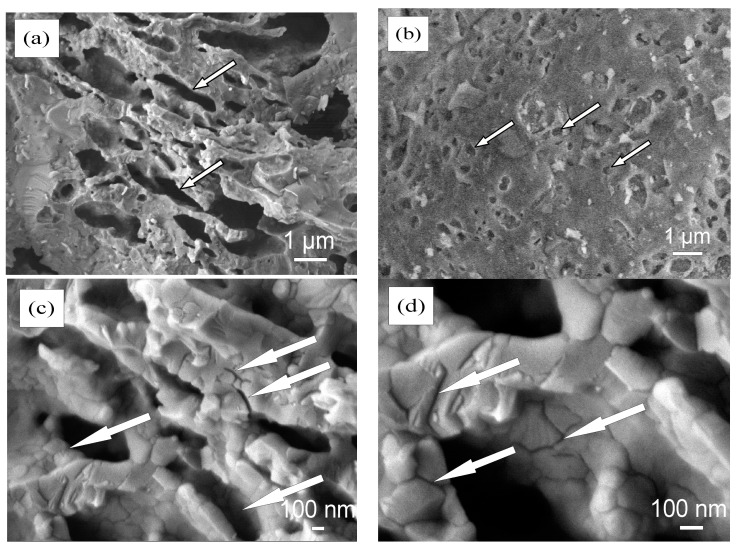
View of the microstructure of ceramic bodies CS and CD20 fired at 1060 °C: (**a**,**b**) magnification ×2000; (**c**) magnification ×40,000; (**d**) magnification ×75,000.

Microstructure analysis of ceramic bodies CS and CD20 fired at 1060 °C showed that burned-out organics in OCW leave elongated and evenly distributed pores of 1–20 µm length and 1.5–5.0 µm width (Figure 8a), suggesting that macropores are not collapsed. This result is proven by previous reports [60,65], where it is pointed out that such a range of macropores remains in the ceramic body after firing. Another source states that elongated pores are common in illite-based ceramic materials [66]. However, in our case, the CS sample is dominated by round-shaped pores which vary in size, in the range of 2–6 µm (Figure 8b). OCW particles are distributed unevenly in the ceramic body (Figure 8c,d). The agglomeration of particles of diatomaceous earth 100–150 nm in diameter is observed. During the firing process, these agglomerated particles melt and form the walls of pores that remain after burning out the organics present in OCW. We can see that on the coarse diatomaceous earth particles some 0.5–1.0 µm wide micro-cracks appeared (Figure 8c), that remain from the burned-out organics, between the particle and the ceramic matrix. The cohesion of particles at their contact zone in pore walls seems to be tight, because, as observed in the image, 10–30 nm wide nano-cracks are not deep, and appeared just on the surface.

### 3.4. XRD Analysis

**Figure 9:**
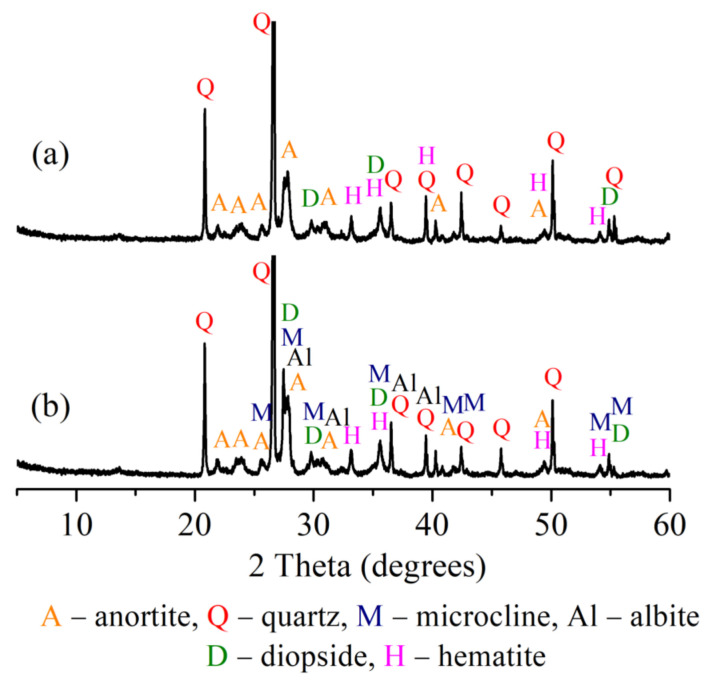
XRD analysis of ceramic bodies fired at 1060 °C: (**a**) CS, (**b**) CD20.

The crystalline phases are developed during sintering at 1060 °C, as can be seen from the XRD curves. XRD curves show that, during decomposition, CaO and MgO oxides react with Al_2_O_3_ and SiO_2,_ originating from the deterioration of the phyllosilicates, to create calcium aluminates and silicates. In the case of composition CS, anorthite, a small amount of diopside, hematite, and quartz (with the most intense peaks) are crystallized (Figure 9). In composition CD20 where the higher amount of alkaline compounds Na_2_O, K_2_O, and alkaline–earth metal compounds such as magnesium and calcium, is present due to the chemical composition of OCW, it can be seen that the available MgO and CaO are involved in diopside (CaMgSi_2_O_6_) formation, which is the main phase formed for this composition. Alkaline compounds Na_2_O and K_2_O appear in new crystalline phases such as microcline (KAlSi_3_O_8_) and albite (NaAlSi_3_O_8_). Similar results for ceramic compositions with diatomaceous earth after firing have been reported [64,67,68,69]. It is also stated that such crystalline phases contribute to the improvement of mechanical strength in ceramic bodies. The intensities of hematite and quartz peaks do not change.

### 3.5. Water Absorption, Apparent Porosity, and Calculated Structural Parameters of Ceramic Samples

Structural parameters help to more fully characterize the pore structure of samples and predict the frost resistance of ceramic samples. The apparent porosity and structural parameters of a ceramic body is an important characteristic that determines its sustainability, and mechanical and thermal conductivity properties [51,70]. Results of water absorption, apparent porosity, and calculated structural parameters of ceramic bodies after firing at different temperatures are presented in Table 5.

**Figure 10:**
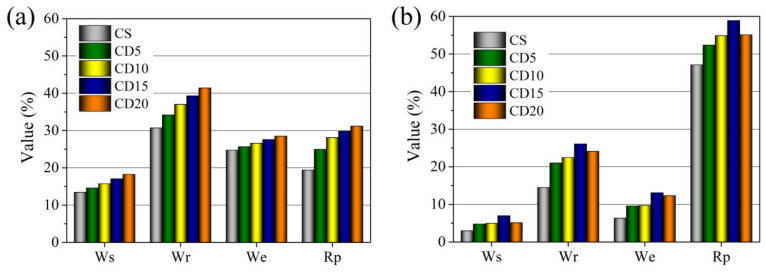
Water absorption (Ws), apparent porosity (Wr), effective porosity (We), and reserve pore volume (Rp), of the ceramic bodies fired at (**a**) 1000 °C and (**b**) 1060 °C.

**Figure 11:**
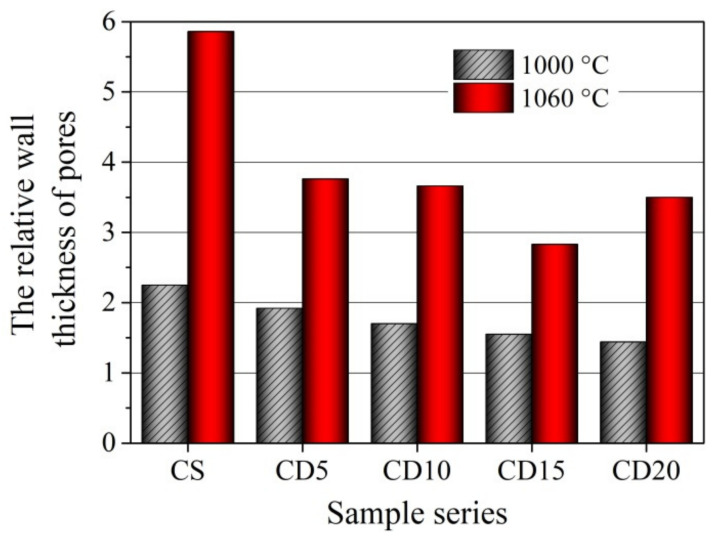
The relative wall thickness of pores in the ceramic samples fired at 1000 °C and 1060 °C temperatures.

Sintering and vitrification processes lead to the growth of open and closed pores, increasing the shrinkage and density of the ceramic body, as well as increasing the formation of new bonds between atoms and the crystal structure in the ceramic body. After burning at 1000 °C, an increase in OCW amount in the formation mix causes an increase in water absorption, apparent porosity, effective porosity, and reserve of porous volume (Figure 10a), while the relative wall thickness of pores in the samples slowly decreases (Figure 11). It can be seen that apparent porosity values in the CD20 sample are approximately 35% higher than in the reference ceramic body because the process of melting in this sample does not reimburse for the growth of apparent porosity attributed to the occurrence of crystalline phases. During firing, organic components in the OCW additive are released during burnout and result in the growth of the effective and apparent porosity of ceramic samples [71]. Additionally, it is noted that OCW contains diatomic earth with a porous structure, which contributes to apparent porosity. The effective porosity of the ceramic body is expressed in the number of effectively working pores and capillaries, whereas the reserve pore volume is the part of the volume into which water is almost unable to penetrate.

Pores fill up slowly when samples are soaked in water and related to the closed damaged plots in the ceramic body, and also on the magnitude of pores and capillaries. The higher the reserve pore space, the better the frost counteraction of the ceramic bodies [54], and higher frost resistance is predicted after firing at 1000 °C for the CD20 ceramic body.

With higher firing temperature and the filling of open pores in the vitrification phase, the apparent porosity and water absorption of ceramic body CS decreases from 30.7 and 13.4%, respectively, after firing at 1000 °C and to 14.5 and 2.9%, after firing at 1060 °C (Figure 10b). Testing of ceramic bodies containing 5–20% of OCW additive revealed that the water absorption and apparent porosity of the ceramic sample depend on OCW waste additive content. With higher OCW waste additive content, the volume of burned-out organics increases, resulting in higher water absorption and apparent porosity. By increasing the OCW composition amount, the apparent porosity and water absorption of specimens increase from 34.18 to 41.4% and from 14.6 to 18.25%, respectively, after firing at 1000 °C. As a result of porous structure formation during firing at 1000 °C, the water absorption of ceramic bodies with OCW is 8.8–36% higher than in reference sample CS. However, increasing the firing temperature to 1040 °C shows, that in the ceramic body with the highest amount of OCW(20%) and, respectively, higher amount of alkali compounds, the apparent porosity and water absorption tend to decrease more, than in the CD15 ceramic body and is by 6.1 and 5.1% less than in the CD15 ceramic body.

After firing at 1060 °C, the apparent porosity and water absorption of ceramic bodies CD5, CD10, and CD15 decreased to 21.01–26.07 and 4.77–6.97%, nearly double that observed in the control ceramic body CS. The microstructure test results showed that pores in ceramic bodies containing OCW additive can cause higher water absorption, than in the ceramic sample without OCW.

The apparent porosity and water absorption of ceramic body CD20 decrease to 24.1 and 5.1%, and it is less by 7.7 and 26.8% than in CD15 ceramic body. That means that the increased volume of burned-out organics does not compensate for the process of melting and vitrification processes leading to decreased pores and structure densifying.

With an increase in firing temperature to 1040 and 1060 °C in the ceramic bodies CS, CD5, CD10, and CD15, the reserve of porous volume and the relative wall thickness of pores increase (Figure 11), especially in the CS body, but the effective porosity decreases (Figure 10b), compared to results after firing at 1000 °C. But the contrary tendency in the ceramic body CD20, other than in ceramic body CD15, is observed. With an increase in firing temperature the reserve of porous volume decreases, but the relative wall thickness of pores increases. This can be explained by the acceleration of the sintering process, intensification of agglomeration, closing of open pores and apparent porosity decreasing. Thicker pore and capillary walls predict greater exploitation of frost resistance and sustainability of ceramic samples. Pores, cavities, and cracks in the ceramic sample with a higher OCW content can be easily saturated with water during freeze–defrost cycles [72]. The appropriate amount of OCW needed to manufacture durable and resistant to freeze-defrost cycles ceramics is 5–10%, according to the study of the physical properties and structural parameters of ceramic samples. Keeping in mind that the lowest shrinkage at 1060 °C is observed in the CD5 and CD10 ceramic bodies, the higher frost resistance can be predicted for this body. Based on the findings in Figure 10b and Figure 11, we can conclude that adding higher amounts (15–20%) of OCW to the composition can insignificantly decrease the sustainability of the ceramic body.

**Figure 12:**
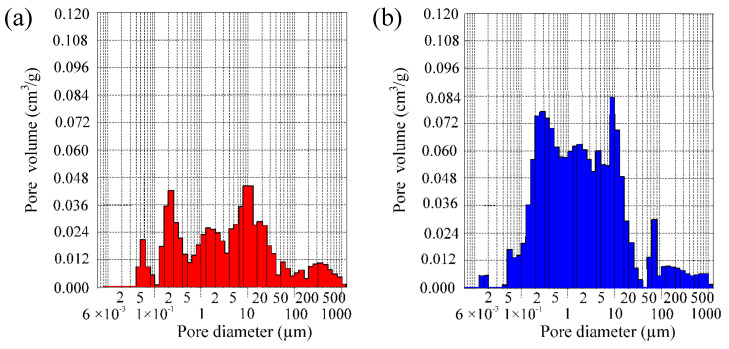
Pore distribution (by volume) in ceramic bodies CS (**a**) and CD20 (**b**) fired at 1060 °C.

The evolution of porosity caused by the firing and combustion at 1060 °C can be seen additionally in the pore size distribution (by volume) of compositions CS and CD20 (Figure 12). The analysis showed that pore size distribution is multimodal. According to mercury porosimeter measurements, in the CS sample, the largest volume of 0.061 cm^3^/g is occupied by pores with diameters ranging from 0.1–40 µm. In sample CD20, the volume of 0.1–20 µm diameter pores increased by 2.2 times, up to 0.1354 cm^3^/g. According to a previous report [64], because of the evolution of crystalline and vitreous phases that progressively seal pores smaller than 0.2 µm, pores smaller than 0.1 µm are practically not observed. This was also confirmed by dilatometric curves, showing shrinkage at this temperature (Figure 6).

According to Maage [53], frost resistance and sustainability of ceramic samples depend upon pore geometry and, specifically, upon the proportion of larger pores, which do not fill up with water readily, to smaller pores, which absorb water that is susceptible to freezing under operating conditions. The Maage factor is based on two relevant factors: the total volume of pores (PV) and the fraction of pores greater than 3 µm (P3). Based on Maage observations and calculations, pores greater than 3 µm in diameter have a positive influence on the frost resistance of bricks. This provision was confirmed elsewhere [73], where the authors established that bricks without any marks of destruction are characterized by a dominance of pores with diameters ranging from 3 to 10 µm. PV and P3 for sample CS are 0.725 cm^3^/g, and 60.4%, and for the sample CD20 sample the values are 1.435 cm^3^/g and 49.5%, respectively. Calculation of Maage factor F_C_ shows values of 144 and 124 for samples CS and CD20, respectively. Both F_C_ values show that these samples are frost resistant, as according to previous work ceramic samples possessing F_C_ values higher than 70 are sustainable [74]; however, the fact that the Fc of sample CS is higher than that of sample CD20 confirms the results of the first structural parameters methodology. It is possible to conclude that the organic matter of OCW during the combustion process increases the volume of 0.1–20 µm diameter pores of the ceramic body, which can positively influence the frost resistance of the ceramic body.

### 3.6. Physical and Mechanical Properties

**Figure 13:**
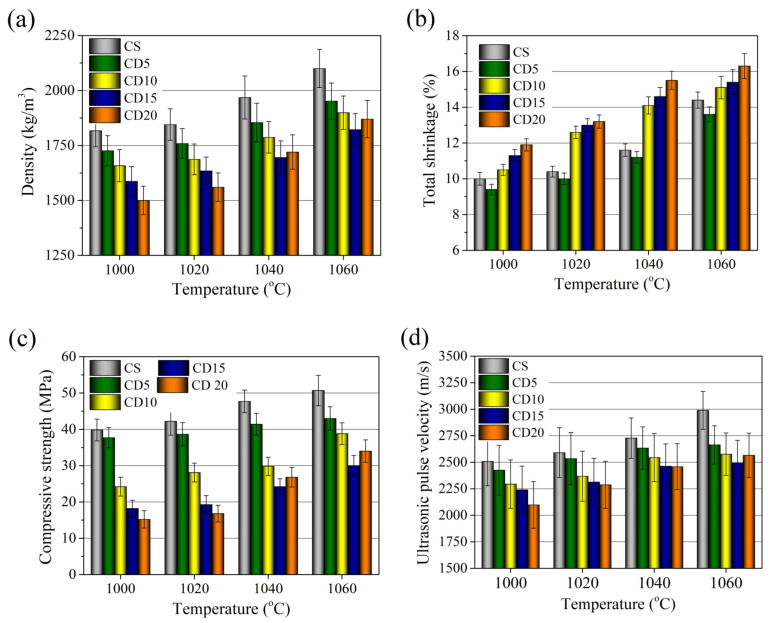
Physical and mechanical properties of the ceramic bodies fired at 1000–1060 °C: (**a**) density, (**b**) total shrinkage, (**c**) compressive strength, (**d**) ultrasonic pulse velocity.

Figure 13 illustrates the density, total shrinkage, compressive strength, and ultrasonic pulse velocity changes of ceramic bodies fired at 1000–1060 °C, respectively. With an increase in firing temperature, the compressive strength and density of the ceramic body CS gradually increase, due to the crystallization process being forced, i.e., the apparent porosity of the ceramic body sharply declines, open pores became closed and shrinkage of the ceramic body grows. Increasing the firing temperature to 1060 °C leads to a much-pronounced growth in sample density (from 1820 kg/m^3^, after firing at 1000 °C, to 2100 kg/m^3^) and in total shrinkage (from 10.0 to 14.4%), respectively. An increase in the compressive strength (from 39.8 to 50.70 MPa) due to compaction of the ceramic body is observed, which is visible from apparent porosity and water absorption values, which declined by a factor of 2.1 and 4.53, respectively. The compressive strength changes are revealed in ultrasonic pulse velocity testing, which shows that, after firing at 1060 °C, ultrasonic pulse velocity increases from 2508 to 2989 m/s in the ceramic body.

Due to the already porous diatomic earth structure and organic compounds creating greater porosity during firing, the microstructure of the ceramic body containing OCW additive has a continuously lower compressive strength and density. Density values of ceramic bodies CD5, CD10, CD15, and CD20 fired at 1000 °C decreased to 1730, 1658, 1570, and 1500 kg/m^3^, respectively, corresponding to 5.2, 8.9, 13.7 and 16.7% lower densities compared to the reference ceramic body.

Increasing OCW amount up to 5% in the forming mixture decreases firing shrinkage down to 9.4%, but higher OCW amounts result in firing shrinkages higher than in the reference ceramic body, reaching 10.5, 11.3, and 11.9%. The compressive strength with an increase in OCW decreases as follows: 37.0, 24.2, 18.2, and 15.2 MPa for CD5, CD10, CD15, and CD20, respectively. The ultrasonic pulse velocity tests support the previously mentioned results, and show a gradual decrease in ultrasonic pulse velocity in the ceramic bodies from 2425 to 2098 m/s, diminishing by 3.3 to 15.9%, respectively, compared to the reference ceramic body. However, it should be noted that in CD5, where the lowest amount of OCW is used, the evolution of the properties (density, compressive strength) is very close to those of the reference CS sample. With an increase in firing temperature up to 1060 °C density values of all compositions of ceramic bodies increase. The addition of OCW changes the technological behavior of the ceramic bodies as follows: as the amount of OCW increases, the ceramic bodies are subject to a substantial decrease in density. Densities values of ceramic bodies CD5, CD10, CD15, and CD20 fired at 1060 °C increased to 1952, 1890, 1790, and 1855 kg/m^3^, respectively, corresponding to 6.2, 9.1, 13.9 and 11.6% lower densities compared to the reference ceramic body. The addition of 20% of OCW due to the highest amount of alkali components and acceleration of the sintering process tends to decrease the number of pores and increases the density.

The compressive strength values vary from 43.0–30.1 MPa for CD5–CD15 ceramic bodies and reach 34 MPa for the CD20 ceramic body. Compressive strengths compared to the reference ceramic body are lower by 15.2, 23.5, 40.8, and 32%.

The increase in density arises from the fact that shrinkage increases with an increase in firing temperature, as can be seen from the dilatometric curve (Figure 6). The growth of the compressive strength of ceramic bodies with OCW can be affected by the crystallization process of newly created minerals, which contributes to an improvement in the mechanical strength of ceramic bodies [68,75]. Also worth noting is that the addition of 5% OCW results in the development of properties at 1060 °C not much different from those of the CS ceramic body, as a constantly lower shrinkage of this ceramic body is observed. In this case, the ceramic body structure compensates for the fusion or vitrification process associated with the occurrence of crystal structures while the temperature is elevated and reflects a higher compressive strength value. In contradistinction, the addition of 10, 15, and 20% OCW leads to lower densities and greater apparent porosities, with a more expressed densification and shrinkage at 1060 °C, especially when 20% of OCW was used. This occurs mainly due to an increased amount of alkaline compounds, with greater tendencies to melting, as can be seen from the dilatometry study (Figure 6). Compared to the CS ceramic body, the compressive strength values remained rather high, and ultrasonic pulse velocity in the CD5, CD10, CD15, and CD20 ceramic bodies is only 10.9–13.8% and 12.1% lower than in the reference ceramic body. However, to gain even lower-density ceramic bodies with adequate mechanical properties, it is feasible to use even 15% of OCW.

### 3.7. Thermal Conductivity

**Figure 14:**
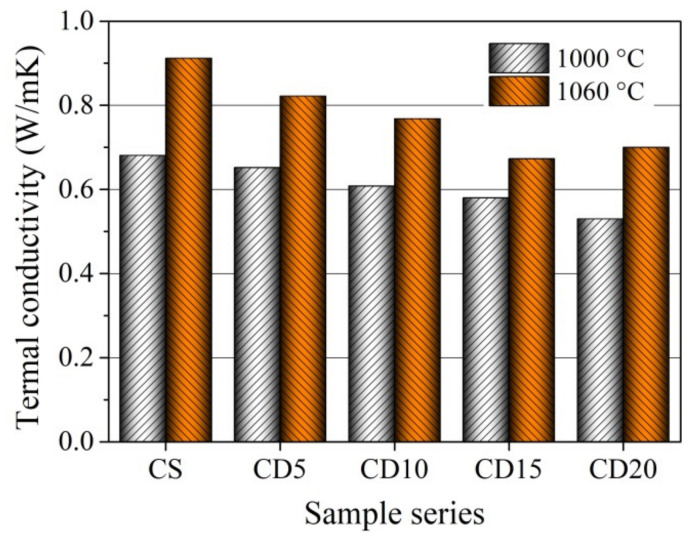
Thermal conductivity of ceramic bodies fired at 1000 and 1060 °C.

The thermal conductivity of the ceramic bodies CS, CD5, CD10, CD15, and CD20 at the two firing temperatures used, is shown in Figure 14. The thermal conductivity of the CS ceramic body (0.68 W/mK after firing at 1000 °C and 0.91 W/mK after firing at 1060 °C) is superior to that of CD5, CD10, CD15, and CD20 ceramic bodies containing OCW. Clay and diatomaceous earth are made of illite group minerals, which have a thermal conductivity of 1.8 W/mK, as well as calcite, forsterite, dolomite, and quartz, with high thermal conductivity values of 3.4, 5.03, 5.51, and 7.7 W/mK, respectively [76]. It is also worth noting that ceramic body CS has a higher quartz content (Table 2), which raises thermal conductivity [66]. The fact that the CS ceramic body has lower apparent porosity is associated with its higher thermal conductivity. Analysis of the thermal conductivity of CD5, CD10, CD15, and CD20 ceramic bodies shows that thermal conductivity results correlate with apparent porosity and density results [29,40,66].

When the OCW amount increases from 5 to 15%, the thermal conductivity of the ceramic body gradually decreases, regardless of the firing temperature used. However, the lowest thermal conductivity of CD15 and CD20 up to 0.58 and 0.53 W/mK after firing at 1000 °C, and 0.67 and 0.70 W/mK after firing at 1060 °C) does not reflect the higher and highest shrinkage obtained by CD15 and CD20, observed in the dilatometric curve (Figure 6) and testing results after firing (Figure 13b). This is related to heterogeneity in the distribution of porous structures (Figure 8 and Figure 12), as well as a rise in the fraction of pores with larger sizes (0.08–20 µm). This happens because as the pore size rises, solid-phase continuity becomes more complicated to achieve, as does heat conduction through the material, which is the major heat transmission mechanism, as well as convection and radiation, which all have a smaller effect [77]. Additionally, it should be noted that the higher amount of alkaline compounds in OCW leads to low-thermal conductivity mineral crystallization [78]. The thermal conductivities of microcline (KAlSi_3_O_8_), albite (NaAlSi_3_O_8_), and diopside (CaMgSi_2_O_6_) are 2.49, 2.14, and 4.23 W/mK. In general, as described in a prior report [33], the addition of OCW decreases energy expenditure throughout the production process. It happens because the energy released during the firing process, as the energy produced from the burning of the organic compounds existing in the diatomaceous earth residue is larger than the extra energy necessary to dry the shaped products.

## 4. Conclusions

According to the findings of this research, the microstructure of the ceramic body containing 10–15% the magnesium silicate/diatomite, contaminated with organic compounds (OCW) had greater porosity after firing at 1000–1060 °C, due to the already-porous diatomic earth structure and organic compounds, resulting in continuous lower densities, compressive strengths, shrinkage, apparent porosity and thermal conductivity value of these ceramic bodies. A higher amount of OCW creates a less porous, denser ceramic body structure, due to an increased amount of alkaline compounds, with greater tendencies to melt. The compressive strength of ceramic bodies with 5, 10, 15, and 20% of OCW additive is lower by 15.2, 23.5, 40.8, and 32% than the reference ceramic body, which is affected by the crystallization of newly created minerals, such as microcline and albite, which contribute to the improvement of mechanical strength in ceramic bodies. It was determined that the organic matter of OCW during the combustion process increases the volume of 0.1–20 µm diameter pores by 2.2 times, which positively influences frost resistance, and decreases thermal conductivity value until 42.5%. According to the calculated structural parameters of ceramic samples, a higher frost resistance is predicted for the ceramic bodies, containing 5 and 10% of OCW. Calculation of the Maage factor (FC) reveals that all ceramic bodies with OCW are frost resistant. These results prove that OCW is a promising material to create value-added, high-strength, sustainable ceramic products that can improve energy efficiency in production and reduce energy consumption in buildings.

## Figures and Tables

**Table 1:** Forming mass compositions.

Raw Materials	Composition of Formation Masses (% of the Mass)				
	CD5	CD10	CD15	CD20	CS
Clay	75	70	65	60	80
Sand	20	20	20	20	20
OCW	5	10	15	20	-

**Table 2:** Chemical compositions of OCW, clay, and sand.

Chemical Composition (%)	OCW	Clay	Sand
SiO_2_	27.4	47.0	90.4
Al_2_O_3_ + TiO_2_	2.50	16.0	4.00
Fe_2_O_3_	1.10	5.31	0.65
CaO	3.50	10.3	2.05
MgO	14.0	4.37	0.49
Na_2_O + K_2_O	4.90	2.50	1.41
Loss on ignition	46.8	14.5	1.02

**Table 3:** Chemical compositions of the forming masses CS, CD5–CD20, calculated by eliminating losses on ignition.

Raw Materials	Chemical Composition (%)					
	SiO_2_	Al_2_O_3_ + TiO_2_	Fe_2_O_3_	CaO	MgO	Na_2_O + K_2_O
CS	63.13	15.42	4.96	9.81	4.07	2.59
CD5	63.16	14.92	4.81	9.60	4.70	2.77
CD10	63.19	14.41	4.65	9.38	5.36	2.97
CD15	63.21	13.87	4.49	9.15	6.04	3.17
CD20	63.23	13.31	4.32	8.91	6.74	3.37

**Table 4:** The calculated temperature at the target viscosity, modulus of acidity, and melting capability modulus.

Forming Masses Mark	Temperature, °C (T_ƞ_)	Modulus of Acidity (M_a_)	Melting Capability Modulus (M_k_)
CS	843	5.66	4.62
CD5	833	5.46	4.02
CD10	827	5.26	3.83
CD15	818	5.07	3.65
CD20	791	4.89	3.48

**Table 5:** Water absorption, apparent porosity, and calculated structural parameters of ceramic samples.

Formation Mix	Ws (%)	Wr (%)	We (%)	Rp (%)	D
After Firing at 1000 °C					
CS	13.42	30.70	24.74	19.41	2.26
CD5	14.60	34.18	25.64	24.93	1.93
CD10	15.72	37.01	26.59	28.10	1.70
CD15	17.05	39.30	27.59	29.79	1.55
CD20	18.25	41.40	28.48	31.19	1.44
After firing at 1020 °C					
CS	12.21	30.59	23.02	24.74	2.27
CD5	12.17	33.35	22.00	34.05	2.00
CD10	13.67	35.99	23.69	34.16	1.78
CD15	14.36	38.29	24.03	37.26	1.61
CD20	14.90	40.29	24.11	38.92	1.49
After firing at 1040 °C					
CS	6.66	21.94	13.37	39.03	3.55
CD5	8.05	26.51	15.35	42.14	2.77
CD10	8.64	29.39	15.91	45.90	2.40
CD15	11.38	34.17	19.81	42.00	1.93
CD20	10.80	32.10	18.10	39.40	2.10
After firing at 1060 °C					
CS	2.96	14.50	6.34	47.10	5.86
CD5	4.77	21.01	9.59	52.35	3.76
CD10	4.93	22.47	9.68	54.88	3.66
CD15	6.97	26.07	13.09	58.90	2.84
CD20	5.10	24.10	12.30	55.1	3.50

## Data Availability

Not applicable.
